# Microperimetry as a diagnostic tool for the detection of early, subclinical retinal damage and visual impairment in multiple sclerosis

**DOI:** 10.1186/s12886-020-01620-9

**Published:** 2020-09-11

**Authors:** Landon J. Rohowetz, Qui Vu, Lilit Ablabutyan, Sean M. Gratton, Nancy Kunjukunju, Billi S. Wallace, Peter Koulen

**Affiliations:** 1 Vision Research Center, Department of Ophthalmology, School of Medicine, University of Missouri – Kansas City, 2411 Holmes St, Kansas City, MO 64108 USA; 2 Harry S Truman Memorial Veterans’ Hospital, Department of Surgery (Ophthalmology section), 800 Hospital Drive, Columbia, MO 65201 USA; 3 Department of Biomedical Sciences, School of Medicine, University of Missouri – Kansas City, 2411 Holmes St, Kansas City, MO 64108 USA

**Keywords:** Multiple sclerosis, Microperimetry, Macular sensitivity

## Abstract

**Background:**

A majority of multiple sclerosis patients experience visual impairment, often as the initial presenting symptom of the disease. While structural changes in the retinal nerve fiber layer and optic nerve have demonstrated correlations with brain atrophy in multiple sclerosis using magnetic resonance imaging, a non-invasive, cost-effective, and clinically efficacious modality to identify early damage and facilitate prompt therapeutic intervention to slow the progression of multiple sclerosis and its ocular manifestations, is still urgently needed. In this study, we sought to determine the role of macular sensitivity measured by microperimetry in the detection of subclinical multiple sclerosis-related retinal damage and visual dysfunction.

**Methods:**

This cross-sectional observational case-control study involved population-based samples of multiple sclerosis patients and age-, race-, and gender-matched healthy control subjects. Among the key criteria for the multiple sclerosis patients were diagnosis by the McDonald criteria, visual acuity greater than 20/25, and no history of optic neuritis. Macular sensitivity and average macular thickness were measured in all subjects using microperimetry and spectral-domain optical coherence tomography, respectively. Pearson correlation coefficients were measured using bivariate correlations. Sample means, mean differences, and 95% confidence intervals were calculated using independent sample t-tests.

**Results:**

Twenty-eight eyes from 14 MS patients and 18 eyes from 9 control subjects were included. Mean macular sensitivity of control subjects and multiple sclerosis patients in decibels was 18.2 ± 0.4 and 16.5 ± 0.4, respectively, corresponding to a mean difference of 1.7 (95% CI, 1.1–2.4; *P* < 0.001). Macular sensitivity was positively correlated with macular thickness in multiple sclerosis patients (r = 0.49, *P* = 0.01) but not control subjects (r = 0.15, *P* = 0.55).

**Conclusions:**

Macular sensitivity as measured by microperimetry was decreased in multiple sclerosis patients with normal visual acuity and no history of optic neuritis. Furthermore, macular sensitivity demonstrated a positive correlation with macular thickness as measured by optical coherence tomography. As such, microperimetry may represent a non-invasive and efficient method to identify signs of subclinical visual dysfunction that correspond with early macular architectural changes characteristic of multiple sclerosis.

## Background

Multiple sclerosis (MS) is an immune-mediated neurodegenerative inflammatory disorder that results in demyelination, axonal dysfunction, gliosis, and ultimately neuronal loss [1–4]. Over 80% of patients with MS experience visual dysfunction and impaired vision often represents the presenting symptom of the disease [5–9]. 73% of individuals with MS experience visual impairment within the first ten years of diagnosis, comparable to the prevalence of motor dysfunction in MS (73–81%) [10]. Although MS frequently presents as reversible symptomatic episodes of neurologic deficits separated in time and space, there is evidence of the presence of ongoing subclinical structural damage [11, 12]. As such, a non-invasive, cost-effective, and clinically efficacious modality to identify damage early and facilitate prompt therapeutic intervention to slow the progression of MS and its ocular manifestations would be invaluable to patients and health care providers.

While many clinical trials have used high-contrast visual acuity as a measure of visual dysfunction, this method may be insufficient to detect subtle visual abnormalities such as loss of color discrimination and low-contrast vision, which are significant contributors to disability in MS. [13] Indeed, several recent studies have identified impaired contrast sensitivity in MS patients with visual acuity of 20/20 or better, illustrating the need for more sensitive methods to detect and monitor visual dysfunction in MS. [5–9, 14–21].

Microperimetry (MP) is an emerging technology that functions as a diagnostic tool by measuring differential light sensitivity of the macular and perimacular regions of the retina while using eye-tracking technology to correct for changes in fixation, thereby eliminating poor fixation as an error factor in examination [22, 23]. MP simultaneously performs scanning laser ophthalmoscopy (SLO) and high-resolution fundus image capture, allowing for integration of MP data and structural parameters. MP is able to efficiently detect localized areas of decreased macular sensitivity [15, 17, 21, 24–26]. However, no peer-reviewed studies have employed MP to assess visual function in MS patients. In this cross-sectional study, we used MP to evaluate macular sensitivity in MS patients with best-corrected visual acuity (BCVA) better than 20/25 and no history of optic neuritis. In addition, we examined macular sensitivity’s relationship with self-reported visual impairment and measures of retinal structure including macular thickness and retinal nerve fiber layer (RNFL) thickness.

## Methods

### Participant selection

Approval for this cross-sectional study was obtained from the Institutional Review Board at the University of Missouri – Kansas City. MS subjects were recruited from neurology and neuro-ophthalmology clinics and an advertisement in the Multiple Sclerosis Society Quarterly newsletter. Age, race, and gender-matched controls were recruited from clinics and flyers. Informed consent for participation was obtained from all subjects.

All testing was performed at the University of Missouri – Kansas City School of Medicine clinical sites in Kansas City, Missouri, USA. Inclusion criteria included age ≥ 18 years, BCVA by Early Treatment Diabetic Retinopathy Study (ETDRS) chart of > 20/25 in the study eye, and intraocular pressure < 21 mmHg. MS patients were required to have been diagnosed by the McDonald criteria [27]. Exclusion criteria included a history of optic neuritis in either eye (verified by medical records, history of suggestive symptoms, or clinical exam showing features of possible optic neuritis), current use of steroids for acute exacerbations, any media opacity affecting retinal sensitivity or preventing capture of good quality scans, diabetes mellitus type 1, uncontrolled diabetes mellitus type II, uncontrolled hypertension, other systemic or neurodegenerative diseases examiner believed may have affected retinal sensitivity or impacted optical coherence tomography (OCT) imaging, evidence of retinopathy or macular pathology, abnormal optic nerve head appearance, any eye that could not be adequately tracked by MP in order to complete the exam, and any eye that could not be adequately scanned to provide sufficient image signal for accurate measurement.

### Sample size

The expected mean for control subjects was determined from a normative database of 19 healthy subjects 40–49 years of age (18.2 ± 1.1 dB) [23]. Since MP has not been measured in MS patients in peer-reviewed studies, the expected mean for MS patients was conservatively predicted at 17.2 ± 1.1 dB. Power was set at 0.80 and α at 0.05. Sample size was calculated at 20 per group using a Cohen’s *d* of 0.91 [28].

### Procedures

Patients were consecutively enrolled. Informed consent was obtained from all patients involved in the study. Visual acuity assessment was measured via high-contrast ETDRS and low-contrast Sloan 2.5% charts. Color vision was assessed using Hardy-Rand-Rittler plates. Applanation was performed using Goldmann tonometry. These measures were collected in all MS patients.

Pupil dilation was obtained using 1% tropicamide and 2.5% phenylephrine drops prior to fundus examination. Macular sensitivity and macular thickness were assessed in all subjects. MP was performed with Optos spectral-domain optical coherence tomography [SD-OCT]/ SLO with add-on Microperimetry module (SLO/SD-OCT microperimeter; Dunfermline, Scotland, UK). Macular sensitivity was recorded at 28 points organized into 3 circles (Polar 3 pattern) using the Optos Microperimetry software. The points were arranged into a central circle with 4 points within 4 degrees from the foveal center, a middle circle with 12 points within 8 degrees, and an outer circle with 12 points within 12 degrees. The target size was 108 μm (Goldman III) and the stimulus duration was 200 milliseconds with a 2 s interval between stimulus presentations. Threshold sensitivity was established at each point using a 4-dB to 2-dB staircase strategy and sensitivity was expressed on a scale of 0 to 20 dB. Fundus localization was automatically tracked by SLO based on retinal vessel alignment.

Measurements of retinal microarchitecture were determined with the Cirrus HD-OCT spectral-domain OCT (Carl Zeiss Meditec, Dublin, CA). Retinal nerve fiber layer (RNFL) thickness and optic nerve head analyses were determined by scanning the optic disc while macular thickness was determined by obtaining a scan centered on the fovea. Retinal thickness was defined in this study as the distance between the RNFL and the retinal pigment epithelial layer. Each scan was manually examined for accuracy of automated layer designation and was repeated as necessary. Retinal thickness was calculated using the ETDRS grid. The grid has a central circle that measures 1 mm in diameter and is centered on the fovea with concentric inner and outer circles that measure 3 mm and 6 mm in diameter, respectively. Central macular thickness was defined as the average thickness within the central circle while average macular thickness was defined as the average thickness within the entire grid. Average macular thickness as measured by Optos SLO/SD-OCT was used to measure agreement with average macular thickness as assessed by Cirrus HD-OCT. All other correlations were obtained using macular thickness as measured by Cirrus HD-OCT.

### Statistical analysis

Patient data was de-identified prior to analysis. Pearson correlation coefficients were measured using bivariate correlations. A Bland-Altman analysis was used to determine the degree of agreement between average macular thickness measured with Optos SLO/SD-OCT and Cirrus HD-OCT. Sample means, mean differences, and 95% confidence intervals were obtained using independent sample t-tests. Hypothesis tests were 2-sided. Repeated measurements were statistically managed by averaging values from both eyes and nonparametric tests (Mann-Whitney U and Spearman’s correlation) were used to confirm statistical significance and degree of correlation. Receiver-operating-characteristic (ROC) curves were calculated by splitting MS patients into two categorical groups of decreased or normal macular thickness based on previously-established normative average and central macular thickness values [29]. Subjects with below-average values were classified as diseased while subjects with above-average values were classified as without disease. The areas under the ROC curves (AUCs) were calculated and compared with the AUC under the reference line. Analyses were performed with Microsoft Excel Version 16.32 (Microsoft Systems, Redmond, WA) and IBM SPSS Statistics Version 25 (IBM Corp., Armonk, NY).

## Results

Twenty-eight eyes from 14 MS patients (12 females, 2 males) and 18 eyes from 9 age- and gender-matched control subjects (6 females, 3 males) were included. The mean age was 48.7 years (SD, 5.6; range, 35–66) for MS patients and 50 years (SD, 7.9; range, 38–57) for control subjects. Groups did not differ in age (*P* = 0.622) or gender (*P* = 0.301). No patients were receiving corticosteroid treatment at the time of the study. All MS patients had perfect color vision by Hardy-Rand-Rittler color plate tests and thus color vision was excluded from the analyses.

Mean macular sensitivity of control subjects and MS patients in decibels was 18.2 ± 0.4 and 16.5 ± 0.4, respectively, corresponding to a mean difference of 1.7 (95% CI, 1.1–2.4; *P* < 0.001). The presence of a cataract did not affect mean macular sensitivity (*P* = 0.16).

**Fig. 1:**
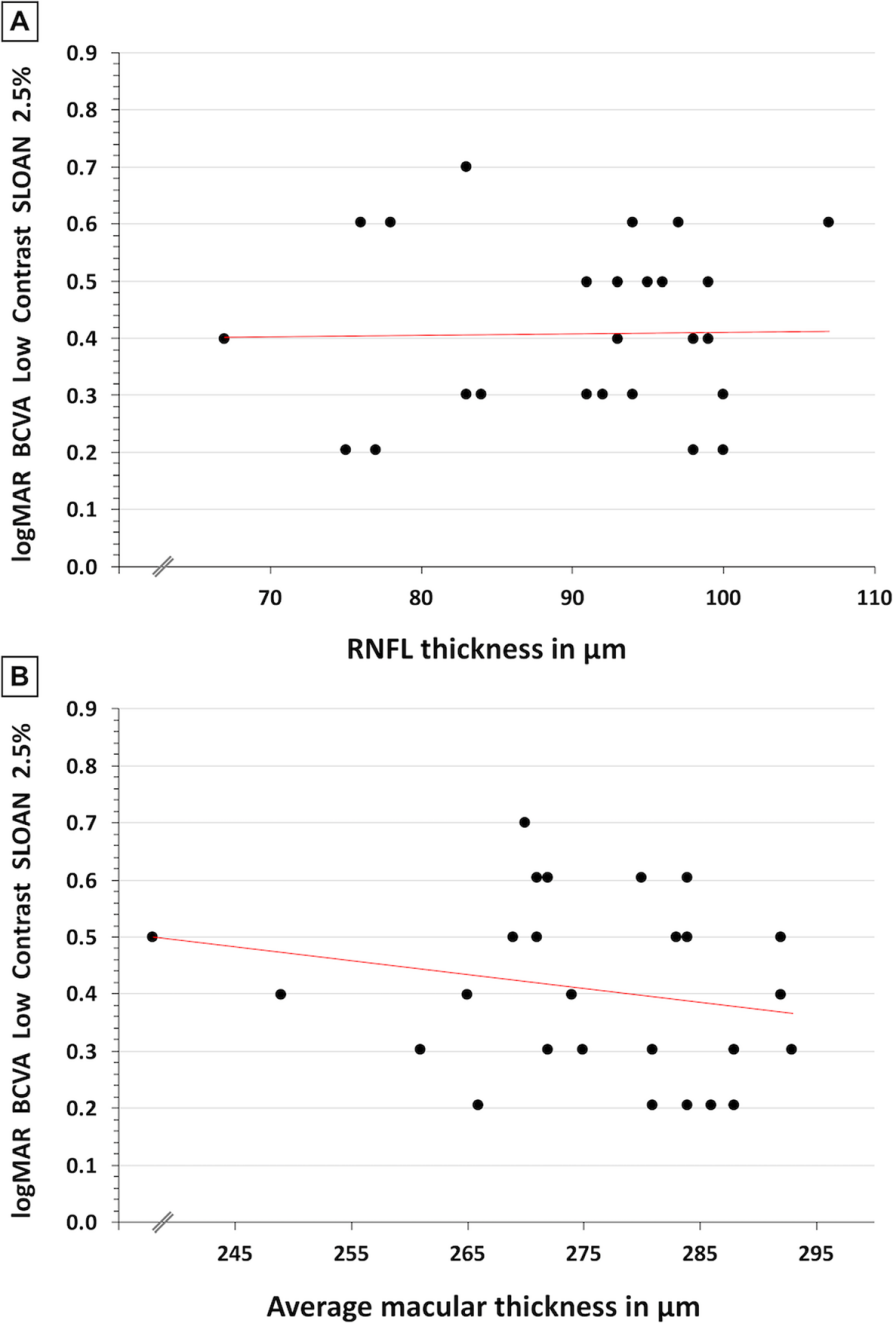
Low-contrast Best-Corrected Visual Acuity. A, Scatterplot demonstrating no correlation (r = − 0.015) between low-contrast best-corrected visual acuity (BCVA) and retinal nerve fiber layer (RNFL) thickness. B, Scatterplot demonstrating no correlation (r = 0.13) between low-contrast BCVA and average macular thickness

Validating results demonstrated by previous studies, our results showed that in MS patients, RNFL thickness measured with OCT is moderately positively correlated with high-contrast BCVA (r = 0.35; *P =* 0.07) [16]. However, no correlation was observed between RNFL thickness and low-contrast BCVA (r = − 0.015; Fig. 1a). Similarly, average macular thickness in MS patients demonstrated a moderately positive correlation with high-contrast BCVA (r = 0.37; *P* = 0.06) but no correlation with low-contrast BCVA (r = 0.13; Fig. 1b).

**Fig. 2:**
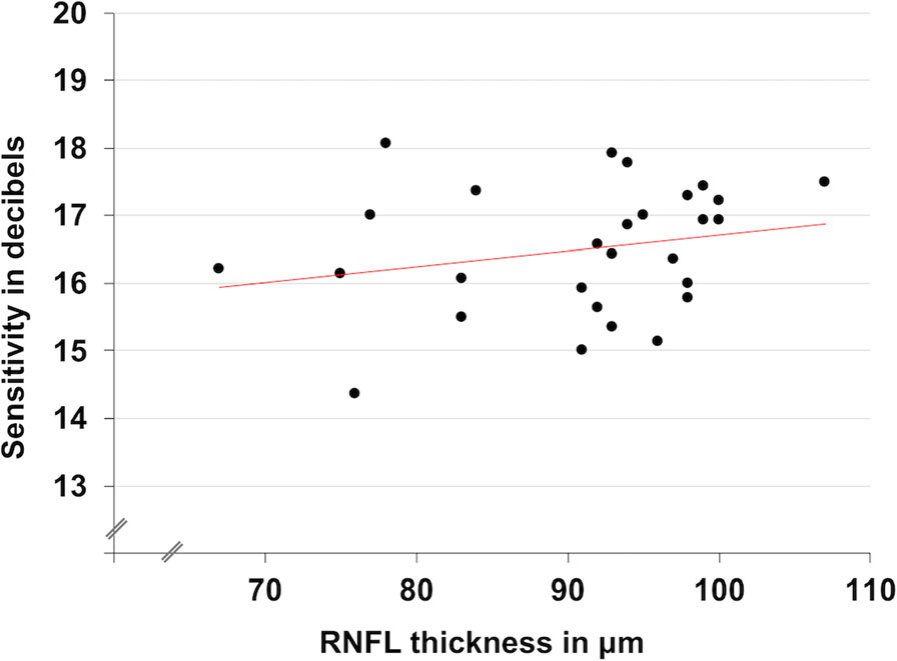
Retinal Nerve Fiber Layer Thickness and Macular Sensitivity. Scatterplot demonstrating moderately positive correlation (r = 0.23, *P* = 0.24) between macular sensitivity and retinal nerve fiber layer (RNFL) thickness in MS patients

**Fig. 3:**
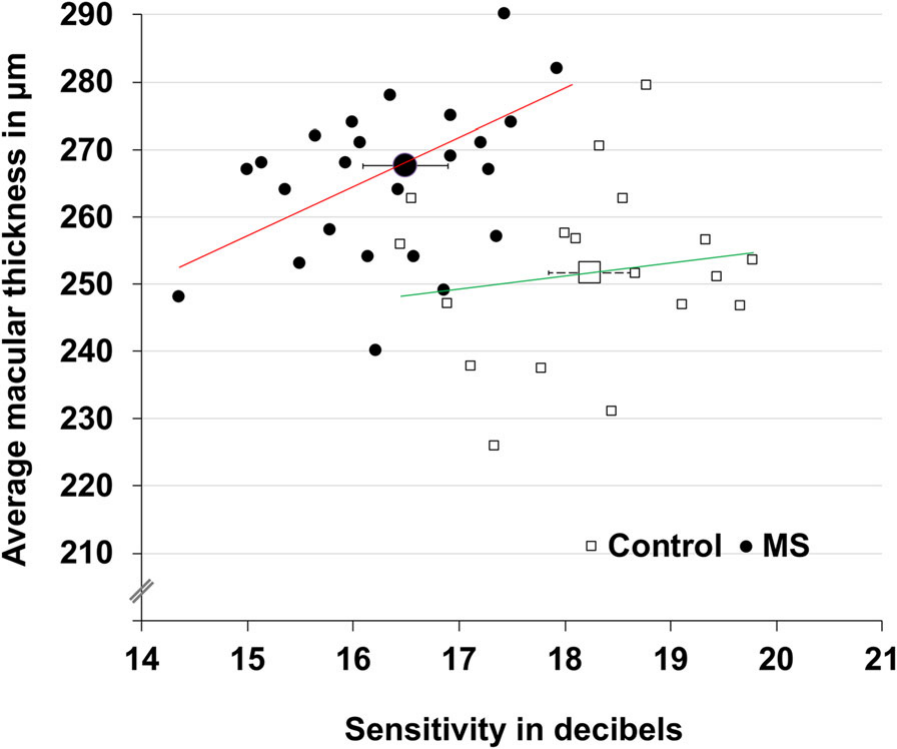
Macular Thickness and Macular Sensitivity. Large square and circle represent mean values of control and multiple sclerosis (MS) groups, respectively. Error bars represent the 95% confidence interval. Mean macular sensitivity in decibels of control subjects and MS patients was 18.2 (95% CI, 17.8–18.7) and 16.5 (95% CI, 16.1–16.8), respectively, corresponding to a mean difference of 1.7 dB (95% CI, 1.1–2.4; *P* < 0.001). Scatterplot demonstrates no relationship between average macular thickness and macular sensitivity in control subjects (r = 0.15) but significant positive correlation in MS patients (r = 0.49, *P* = 0.01)

Bland-Altman analysis of average macular thickness measured with Optos SLO/SD-OCT and Cirrus HD-OCT demonstrated significant agreement with a mean difference of 7.23 μm (95% CI, − 15.67 to 30.14 μm). Macular sensitivity measured with MP was moderately positively correlated with RNFL thickness (r = 0.23; *P* = 0.24; Fig. 2). While in control subjects there was no association (r = 0.15), MS patients demonstrated a significant positive association between macular sensitivity and central (r = 0.51; *P* = 0.006) and average (r = 0.49; *P* = 0.01; Fig. 3) macular thickness. Furthermore, ROC curves evaluating the ability of MP to identify MS patients with decreased average and central macular thickness revealed AUCs of 0.842 (95% CI, 0.689 to 0.994; *P* < 0.001; Figure S1) and 0.741 (95% CI, 0.533 to 0.950; *P* = 0.023; Figure S2), respectively, while ROC curves evaluating the ability of low-contrast BCVA to identify the aforementioned patient populations revealed AUCs of 0.663 (95% CI, 0.460 to 0.867, *P* = 0.116; Figure S3) and 0.418 (95% CI, 0.212 to 0.625; *P* = 0.439; Figure S4), respectively.

## Discussion

In the present study, we provide clinical evidence that macular sensitivity measured with MP is decreased in MS patients with normal visual acuity and no history of optic neuritis. Our results also reveal a significant correlation between macular thickness and macular sensitivity in MS patients, a relationship that is not seen in control subjects (Fig. 3). As such, MP may represent a relatively novel technique to identify visual dysfunction and monitor disease progression in MS patients with visually-predominant symptoms.

Low-contrast BCVA has been used to detect retinal damage and visual dysfunction in MS patients with normal high-contrast BCVA. However, bivariate correlations from the current study demonstrate no relationship between low-contrast BCVA and measures of retinal microarchitecture (Fig. 1a and b). Furthermore, ROC analyses suggest that low-contrast BCVA may lack adequate sensitivity to detect early retinal damage associated with MS (Figures S3 and S4). On the other hand, macular sensitivity demonstrates correlations with architectural parameters affected in MS (Figs. 2 and 3) and MP demonstrates significant diagnostic accuracy for detecting patients with decreased average and central macular thickness (Figure S1 and S2). As such, MP may be more sensitive than low-contrast vision charts in assessing visual pathway damage caused by MS.

MP assesses macular function and MS preferentially affects the central macula [23, 30]. The most significant structural relationships in this study were seen with macular sensitivity and central and average macular thickness (Fig. 3). However, peripapillary RNFL thickness is also decreased in MS and in this study demonstrated a moderately positive correlation with macular sensitivity (Fig. 2) [31]. Therefore, while most valuable for evaluating macular damage, MP may also serve as a marker for generalized retinal damage.

Indeed, macular sensitivity and thickness in MS patients with normal or near-normal visual acuity appear to be superior indicators of early subclinical retinal damage and visual impairment associated with the disease when compared to other functional and structural parameters. These measurements are particularly valuable insofar that they can be obtained simultaneously and non-invasively within minutes and can be used to detect objective signs of early visual dysfunction. Moreover, these measures may be more sensitive in detecting visual dysfunction than self-reported measures, similar to objective measurements obtained via OCT that have been shown to demonstrate axonal damage in the absence of subjective findings in MS. [31] As such, macular sensitivity may be used in conjunction with macular thickness to identify damage to the visual pathway before visual impairments are detected by patients. This valuable information may allow physicians to make more appropriate and informed treatment decisions to prevent and slow disease progression.

While significant findings were observed in this study, the sample size *(n* = 28 MS eyes*; n* = 18 control eyes*)* was relatively small. Further investigation should seek to increase sample size to further stratify patient populations based on disease-specific and potentially other medically relevant criteria. Future studies may also seek to further evaluate the utility of monitoring macular sensitivity and thickness in MS by observing longitudinal changes in these parameters and their correlation with disease progression as compared to other available subjective and objective measures. Furthermore, as optic neuritis is a prevalent symptom in MS, subsequent analysis should seek to evaluate the potential of using macular sensitivity and macular thickness as indicators of disease activity in MS patients with a history of optic neuritis.

## Conclusions

Diagnostic modalities that detect and monitor for signs of MS-related visual dysfunction are essential to guiding decisions regarding treatment and prevention. This is the first peer-reviewed study to assess the utility of MP in the evaluation of MS-associated visual dysfunction. In this study, we present MP as a novel method to detect early visual dysfunction in MS that correlates with concurrent microarchitectural changes associated with the disease. Findings from this study provide a valid rationale for the incorporation of MP combined with OCT into the routine clinical assessment of MS patients. Furthermore, our results illustrate the possibility of using these technologies in the evaluation of novel, potentially vision-saving treatments in future clinical trials designed to preserve visual function and quality of life in individuals affected by MS.

## Supplementary information

**Additional file 1: Figure S1.** Diagnostic accuracy of microperimetry for detecting decreased average macular thickness. **Figure S2.** Diagnostic accuracy of microperimetry for detecting decreased central macular thickness. **Figure S3.** Diagnostic accuracy of low-contrast BCVA for detecting decreased average macular thickness. **Figure S4.** Diagnostic accuracy of low-contrast BCVA for detecting decreased central macular thickness.

## Data Availability

The datasets used and analyzed during the current study are available from the corresponding author on reasonable request.

## Abbreviations

BCVA: Best-corrected visual acuity
ETDRS: Early Treatment Diabetic Retinopathy Study
MP: Microperimetry
MS: Multiple sclerosis
OCT: Optical coherence tomography
RNFL: Retinal nerve fiber layer thickness
ROC: Receiver-operating-characteristic
SD-OCT: Spectral-domain optical coherence tomography
SLO: Scanning laser ophthalmoscopy
