# Supplementary material for: Microperimetry as a diagnostic tool for the detection of early, subclinical retinal damage and visual impairment in multiple sclerosis
Source: BMC Ophthalmol. 2020 Sep 11;20:367. doi: 10.1186/s12886-020-01620-9 (PMC7488495; doi:10.1186/s12886-020-01620-9)
Supplement: Supplementary file 1 — Additional file 1: Figure S1. Diagnostic accuracy of microperimetry for detecting decreased average macular thickness. Figure S2. Diagnostic accuracy of microperimetry for detecting decreased central macular thickness. Figure S3. Diagnostic accuracy of low-contrast BCVA for detecting decreased average macular thickness. Figure S4. Diagnostic accuracy of low-contrast BCVA for detecting decreased central macular thickness. [file 12886_2020_1620_MOESM1_ESM.docx]

Figure S1.

**Diagnostic accuracy of microperimetry for detecting decreased average macular thickness.**

Receiver-operating-characteristic (ROC) curve illustrating the diagnostic performance of microperimetry for detecting decreased average macular thickness in multiple sclerosis patients with normal high-contrast best-corrected visual acuity. The area under the ROC curve (0.842 [95% CI, 0.689 to 0.994]) is significantly greater than the area under the reference line (0.5; *P* < 0.001).

Figure S2.

**Diagnostic accuracy of microperimetry for detecting decreased central macular thickness.**

Receiver-operating-characteristic (ROC) curve illustrating the diagnostic performance of microperimetry for detecting decreased central macular thickness in multiple sclerosis patients with normal high-contrast best-corrected visual acuity. The area under the ROC curve (0.741 [95% CI, 0.533 to 0.950]) is significantly greater than the area under the reference line (0.5; *P* = 0.023).

Figure S3.

**Diagnostic accuracy of low-contrast BCVA for detecting decreased average macular thickness.**

Receiver-operating-characteristic (ROC) curve illustrating the diagnostic performance of low-contrast BCVA for detecting decreased average macular thickness in multiple sclerosis patients with normal high-contrast best-corrected visual acuity. The area under the ROC curve (0.663 [95% CI, 0.460 to 0.867]) is not significantly different than the area under the reference line (0.5; *P* = 0.116).

Figure S4.

**Diagnostic accuracy of low-contrast BCVA for detecting decreased central macular thickness.**

Receiver-operating-characteristic (ROC) curve illustrating the diagnostic performance of low-contrast BCVA for detecting decreased central macular thickness in multiple sclerosis patients with normal high-contrast best-corrected visual acuity. The area under the ROC curve (0.418 [95% CI, 0.212 to 0.625]) is not significantly different than the area under the reference line (0.5; *P* = 0.439).
